# Supplementary material for: High‐resolution mapping of cancer cell networks using co‐functional interactions
Source: Mol Syst Biol. 2018 Dec 20;14(12):e8594. doi: 10.15252/msb.20188594 (PMC6300813; doi:10.15252/msb.20188594)
Supplement: Supplementary file 1 — Expanded View Figures PDF [file MSB-14-e8594-s001.pdf]

Expanded View Figures

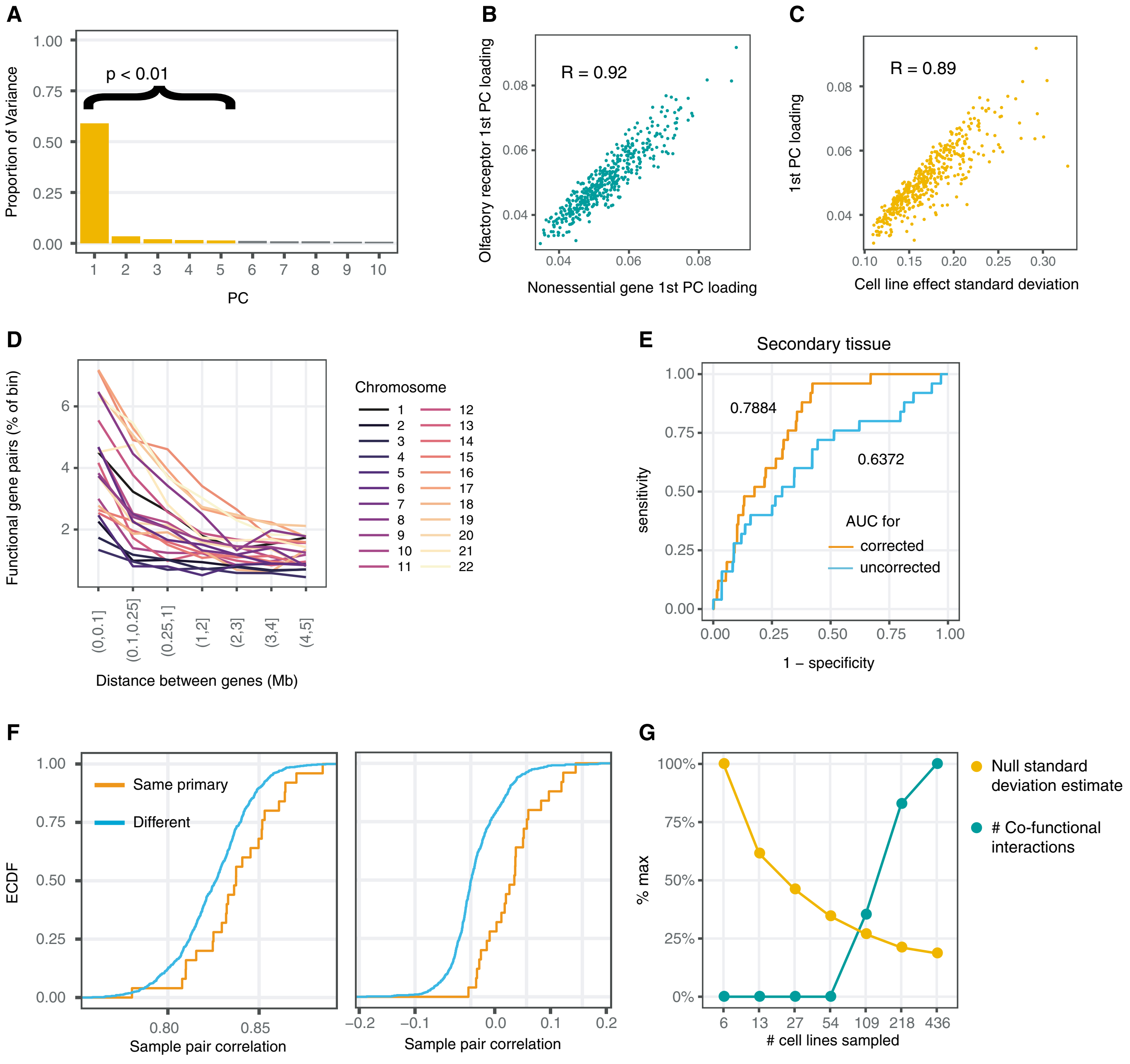

Figure EV1. Characterization of the correction for gene essentiality profiles.

A PCA on olfactory receptors identifies 5 significant principal components by permutation, with most variance explained in the first principal component. Permutation test as described in Materials and Methods.

B Agreement of first principal component loadings calculated using olfactory receptors (*y*) and curated nonessential genes (*x*).

C, D The rate of calling co-functional gene pairs varies by chromosome and physical distance.

E Area under the receiver operating characteristic (ROC) curve for detecting two samples of the same primary disease.

F The empirical distributions of cell line profile pairwise correlations before and after regressing the five principal components from (A) differ in mean.

G Downsampling analysis of number of co-functional interactions and estimated null standard deviation relative to the full dataset (436) as a function of the number of cell line screens used.

© 2018 The Authors

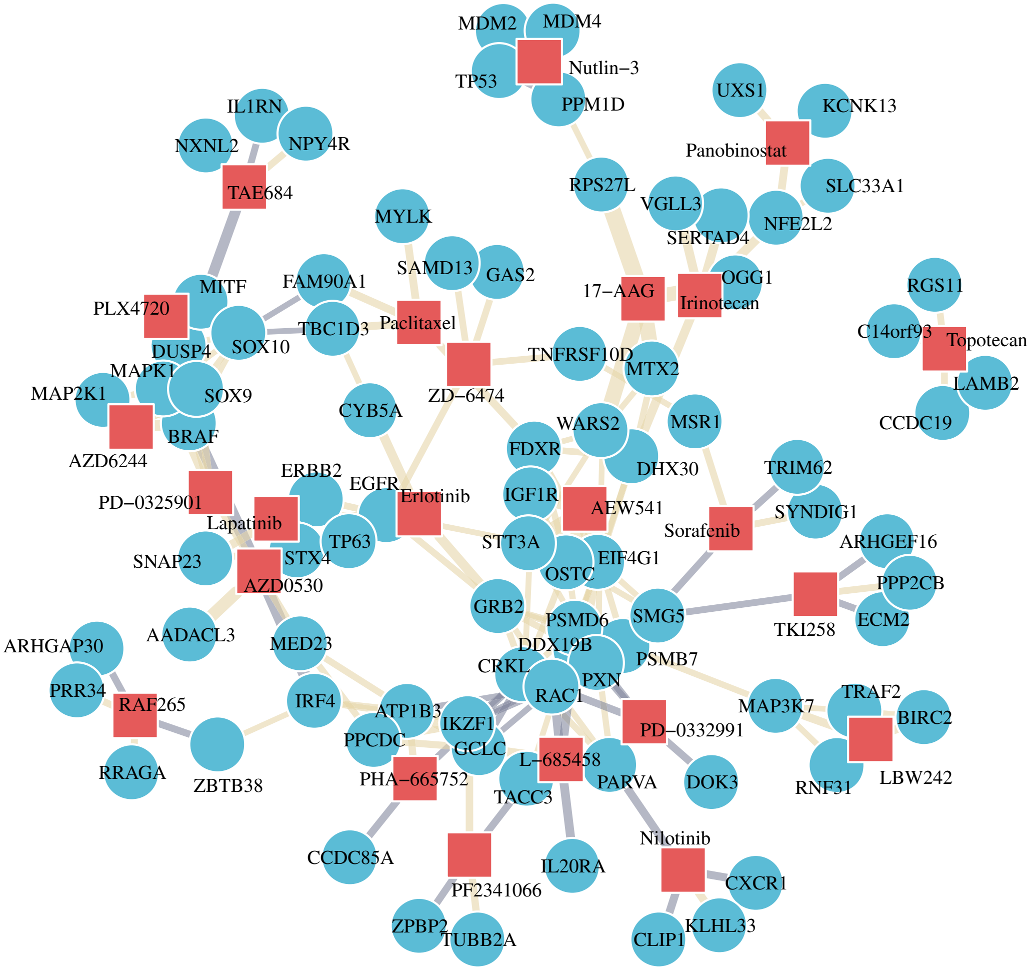

Figure EV2. Drug–gene network.

Drugs (red squares) and their most correlated genes (blue circles) by corrected gene essentiality profiles. Correlations between gene profiles and drug profiles across cell lines create biologically meaningful clusters for drug targets such as ErbB family members and MAPK pathways.

Figure EV3. Properties of the co-functional gene network with respect to gene annotation databases.

A Enrichment of disconnected genes in the network across Gene Ontology Biological Process gene sets highlights terms specifying tissue differentiation and development.

B Co-functionality rates binned by both STRING interaction type and confidence level. “xferred” (transferred) reflects associations mapped from non-human organisms.

C Co-functionality rates for genetic interactions annotated with a detailed interaction type in STRING, as in (A).

D Percent of co-functional gene edges that derived from negatively correlated corrected gene essentiality profiles, binned as in (C).

E Density of pairs of genes participating in CORUM core complexes by mean knockout phenotype of each gene. Gene pairs that are not called as co-functional are on the left; validated gene pairs are on the right. Gene pairs with no growth deficit upon knockout are generally not called as co-functional.

 © 2018 The Authors

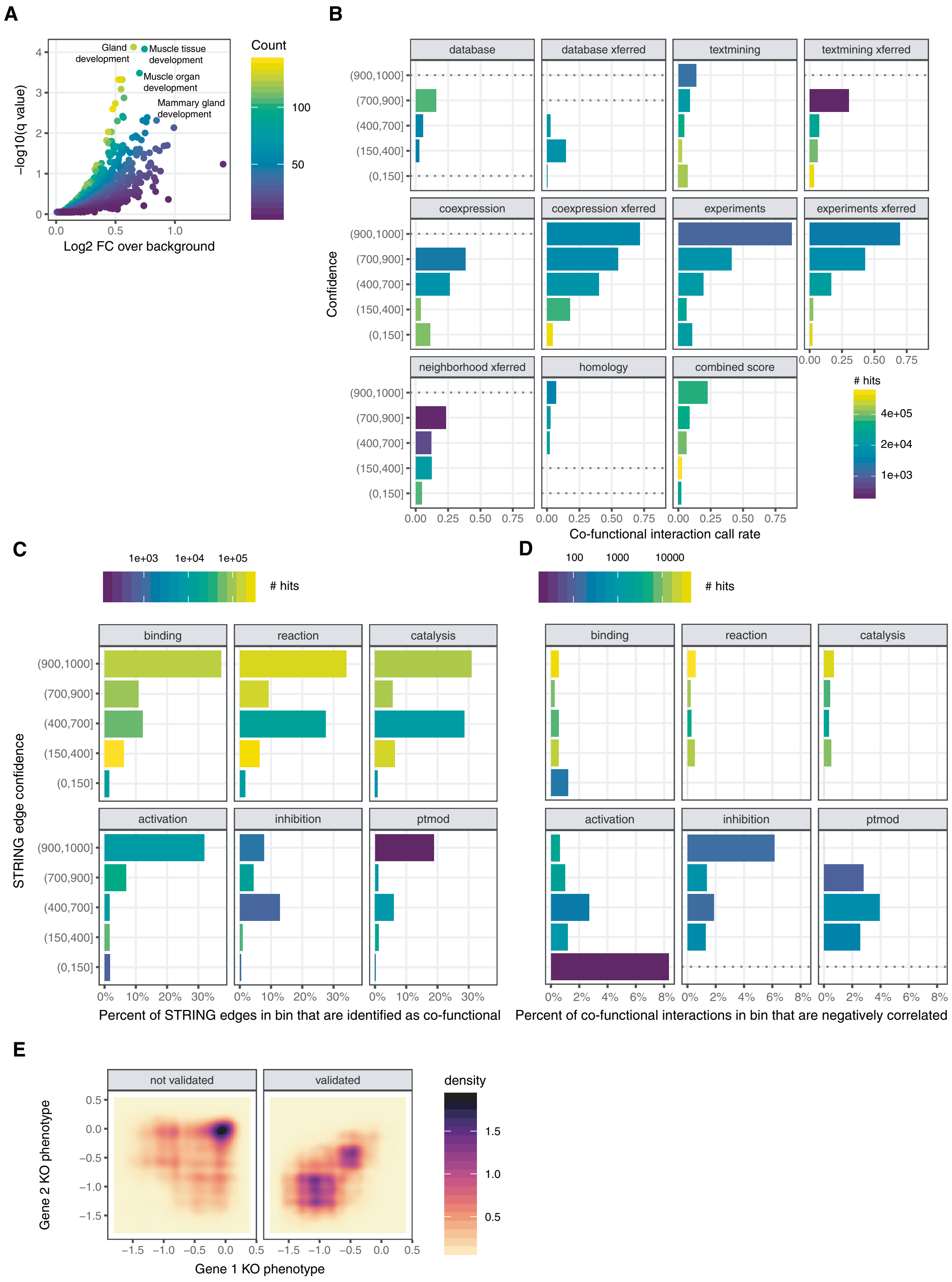
A
Count
100
50
Gland development
Muscle tissue development
Muscle organ development
Mammary gland development
−log10(q value)
Log2 FC over background
B
database
database xferred
textmining
textmining xferred
coexpression
coexpression xferred
experiments
experiments xferred
neighborhood xferred
homology
combined score
(900,1000]
(700,900]
(400,700]
(150,400]
(0,150]
Confidence
hits
4e+05
2e+04
1e+03
Co-functional interaction call rate
C
1e+03 1e+04 1e+05
hits
binding
reaction
catalysis
activation
inhibition
ptmod
STRING edge confidence
Percent of STRING edges in bin that are identified as co-functional
D
100 1000 10000
hits
Percent of co-functional interactions in bin that are negatively correlated
E
not validated
validated
density
Gene 2 KO phenotype
Gene 1 KO phenotype

Figure EV3.

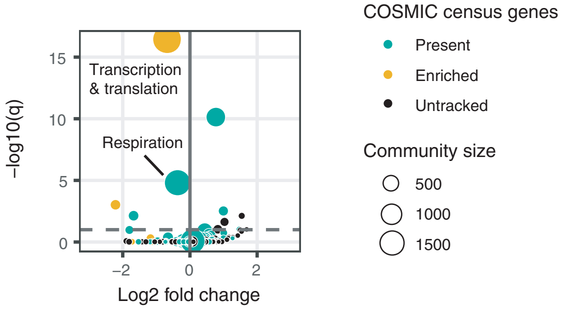

Figure EV4. Uncharacterized genes in the cancer cell network.

Volcano plot of uncharacterized gene enrichment in gene communities. Gold nodes are gene communities containing more COSMIC cancer census genes than expected by random assortment. Green nodes are gene communities containing at least one COSMIC cancer census gene.

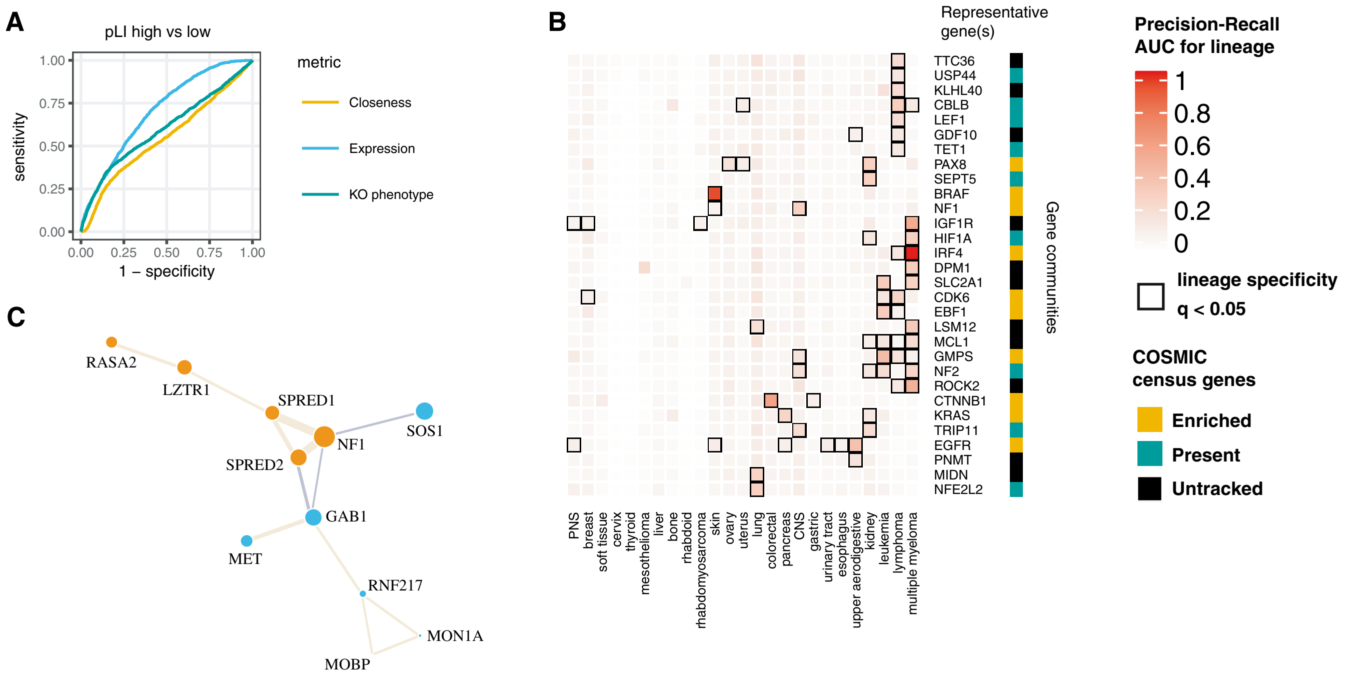

Figure EV5. Variation in topology and cell type specificity of gene communities.

A ROC curve showing that loss of function-intolerant (high pLI) genes are distinguished by high expression and only weakly by growth knockout phenotype and network topology.

B Community scores calculated de novo from the co-functionality network often describe cell type-specific cancer regulation, especially true for gene communities enriched for COSMIC census genes (in gold).

C Full depiction of the NF1 gene community. Size of the gene nodes reflects loading on the first principal component among community members.

 © 2018 The Authors
